# Supplementary material for: Association between Parents’ Perceptions of Preschool Children’s Weight, Feeding Practices and Children’s Dietary Patterns: A Cross-Sectional Study in China
Source: Nutrients. 2021 Oct 25;13(11):3767. doi: 10.3390/nu13113767 (PMC8624819; doi:10.3390/nu13113767)
Supplement: Supplementary file 1 [file nutrients-13-03767-s001.zip › nutrients-1356377-supplementary.pdf]

Table S1. Linear regression relationships between parental weight perceptions and children's dietary patterns among different ZBMI weight status of children

Weight Perceptions	Traditional Dietary Pattern			Snacking Dietary Pattern		
	$\hat{\beta}$	t	*P*	$\hat{\beta}$	t	*P*
**Underweight**						
**Concern about child becoming overweight**						
Unconcerned	Ref	-	-	Ref	-	-
Concerned	-0.045	-0.653	0.514	**0.187**	**2.711**	**0.007**
**Satisfaction with child body weight**						
Same	Ref	-	-	Ref	-	-
Wish thinner	**-0.146**	**-2.013**	**0.045**	**0.162**	**2.195**	**0.029**
Wish heavier	**-0.189**	**-2.619**	**0.009**	-0.024	-0.322	0.648
**Misperception of child body weight**						
Correct	Ref	-	-	Ref	-	-
Overestimate	**0.156**	**2.343**	**0.020**	0.041	0.603	0.547
**Normal weight**						
**Concern about child becoming overweight**						
Unconcerned	Ref	-	-	Ref	-	-
concerned	0.041	1.277	0.220	0.015	0.453	0.650
**Satisfaction with child body weight**						
Same	Ref	-	-	Ref	-	-
Wish thinner	-0.024	-0.660	0.509	0.040	1.099	0.272
Wish heavier	-0.064	-1.765	0.078	0.040	1.110	0.267
**Misperception of child body weight**						
Correct	Ref	-	-	Ref	-	-
Overestimate	0.035	1.043	0.297	0.026	0.786	0.432
Underestimate	-0.051	-1.535	0.125	-0.029	-0.870	0.385
**Overweight/Obese**						
**Concern about child becoming overweight**						
Unconcerned	Ref	-	-	Ref	-	-
Concerned	-0.056	-1.047	0.296	0.079	1.492	0.137
**Satisfaction with child body weight**						
Same	Ref	-	-	Ref	-	-
Wish thinner	-0.067	-1.124	0.262	0.003	0.507	0.955
Wish heavier	0.002	0.026	0.980	-0.078	-1.333	0.183
**Misperception of child body weight**						
Correct	Ref	-	-	Ref	-	-
Underestimate	0.002	0.034	0.973	0.073	1.358	0.173

$\hat{\beta}$, Standardized Regression coefficients.
Regression model with adjustments of child gender, age, only child, parental role and parental education.

Table S2. Linear regression relationships between parental weight perceptions and caregiver feeding practices among different ZBMI weight status of children

Weight Perceptions	Restriction			Food as Reward			Pressure to Eat			Monitoring		
	$\hat{\beta}$	t	*P*	$\hat{\beta}$	t	*P*	$\hat{\beta}$	t	*P*	$\hat{\beta}$	t	*P*
**Underweight**												
**Concern about child becoming overweight**												
Unconcerned	Ref	-	-	Ref	-	-	Ref	-	-	Ref	-	-
Concerned	**-0.141**	**-2.011**	**0.046**	-0.080	-1.144	0.254	-0.075	-1.082	0.280	**-0.152**	**-2.204**	**0.029**
**Satisfaction with child body weight**												
Same	Ref	-	-	Ref	-	-	Ref	-	-	Ref	-	-
Wish thinner	0.030	0.396	0.693	-0.013	-0.170	0.865	-0.029	-0.403	0.687	0.017	0.225	0.822
Wish heavier	0.122	1.635	0.104	0.122	1.641	0.102	**0.250**	**3.506**	**0.001**	0.118	1.603	0.110
**Misperception of child body weight**												
Correct	Ref	-	-	Ref	-	-	Ref	-	-	Ref	-	-
Overestimate	-0.103	-1.507	0.133	-0.089	-1.297	0.196	-0.127	-1.893	0.060	-0.077	-1.143	0.254
**Normal weight**												
**Concern about child becoming overweight**												
Unconcerned	Ref	-	-	Ref	-	-	Ref	-	-	Ref	-	-
Concerned	**0.071**	**2.138**	**0.033**	0.001	0.037	0.971	0.015	0.454	0.650	0.018	0.529	0.597
**Satisfaction with child body weight**												
Same	Ref			Ref			Ref			Ref		
Wish thinner	-0.003	-0.082	0.935	-0.008	-0.231	0.817	-0.043	-1.208	0.227	0.006	0.166	0.868
Wish heavier	-0.061	-1.669	0.095	0.012	0.321	0.748	**0.115**	**3.227**	**0.001**	-0.006	-0.173	0.863
**Misperception of child body weight**												
Correct	Ref			Ref			Ref			Ref		
Overestimate	0.054	1.616	0.106	0.043	1.286	0.199	0.021	0.627	0.531	0.053	1.613	0.107
Underestimate	-0.053	-1.600	0.110	0.023	0.678	0.498	**0.079**	**2.399**	**0.017**	0.019	0.563	0.573
**Overweight/Obese**												
**Concern about child becoming overweight**												
Unconcerned	Ref	-	-	Ref	-	-	Ref	-	-	Ref	-	-
Concerned	0.027	0.499	0.618	-0.064	-1.194	0.233	-0.075	-1.416	0.158	-0.016	-0.301	0.763
**Satisfaction with child body weight**												
Same	Ref	-	-	Ref	-	-	Ref	-	-	Ref	-	-
Wish thinner	0.100	1.690	0.092	-0.007	-0.113	0.910	-0.019	-0.326	0.745	0.090	1.517	0.130
Wish heavier	-0.041	-0.696	0.487	-0.029	-0.494	0.622	**0.170**	**2.940**	**0.004**	0.069	1.183	0.238
**Misperception of child body weight**												
Correct	Ref	-	-	Ref	-	-	Ref	-	-	Ref	-	-
Underestimate	**-0.108**	**-2.003**	**0.046**	0.025	0.468	0.640	**0.168**	**3.152**	**0.002**	-0.046	-0.856	0.393

$\hat{\beta}$, Standardized Regression coefficients.
Regression model with adjustments of child gender, age, only child, parental role and parental education.

Table S3. Linear regression relationships between parental feeding practices and children's dietary patterns among different ZBMI weight status of children

**Weight Perceptions**	**Traditional Dietary Pattern**			**Snacking Dietary Pattern**		
	$\hat{\beta}$	**t**	***P***	$\hat{\beta}$	**t**	***P***
**Underweight**						
Restriction	0.044	0.574	0.567	**-0.189**	**-2.531**	**0.012**
Food as reward	-0.001	-0.012	0.991	-0.033	-0..494	0.622
Pressure to eat	**-0.151**	**-2.202**	**0.029**	0.038	0.572	0.568
Monitoring	**0.194**	**2.542**	**0.012**	**-0.208**	**-2.792**	**0.006**
**Normal weight**						
Restriction	0.001	0.015	0.988	**-0.091**	**-2.385**	**0.017**
Food as reward	**-0.072**	**-2.083**	**0.038**	-0.041	-1.197	0.232
Pressure to eat	0.009	0.270	0.787	**0.090**	**2.677**	**0.008**
Monitoring	**0.104**	**2.647**	**0.008**	**-0.143**	**-3.745**	**<0.001**
**Overweight/Obese**						
Restriction	0.010	0.145	0.885	**-0.221**	**-3.527**	**<0.001**
Food as reward	-0.065	-1.153	0.250	-0.071	-1.330	0.185
Pressure to eat	0.015	0.271	0.787	0.060	1.139	0.255
Monitoring	**0.154**	**2.405**	**0.017**	-0.106	-1.724	0.086

$\hat{\beta}$, Standardized Regression coefficients.
Regression model includes restriction, food as reward, pressure to eat and monitoring, plus child gender, age, only child, parental role and parental education.
